# Phenolation to Improve Hardwood Kraft Lignin for Wood Adhesive Application

**DOI:** 10.3390/polym16131923

**Published:** 2024-07-05

**Authors:** Li-Yuan Liu, Wan-Shuan Chiang, Hou-min Chang, Ting-Feng Yeh

**Affiliations:** 1 School of Forestry and Resource Conservation, National Taiwan University, Taipei 106, Taiwan; yuan79218@gmail.com (L.-Y.L.); e1f3e8f2@gmail.com (W.-S.C.); 2 Department of Forest Biomaterials, North Carolina State University, Raleigh, NC 27695, USA; hchang@ncsu.edu

**Keywords:** adhesive, hardwood kraft lignin, phenolation, phenol formaldehyde resin, plywood

## Abstract

Lignins, naturally occurring aromatic polymers with phenylpropane units, are promising bio-based alternatives for petroleum-based products. Resole-type phenol formaldehyde (PF) adhesive is commonly used in wood composites requiring durability and weather-proofness. However, PF adhesive is a petroleum-based product. The objective of this study is to transform the low-reactivity hardwood kraft lignin (KL) as the phenol substitute in the PF adhesive formulation by acidic phenolation. The variations in the molecular weights, chemical structures, and functional groups in lignins were investigated before and after the phenolation. The results indicate that the KL can be cleaved, and phenols are crosslinked onto KL to produce phenolated kraft lignin (PKL) under the suitable phenolation condition, heating 3/5 (*w*/*w*) of KL/phenol at 90 °C for 2 h with 5% H_2_SO_4_ as the catalyst. Resole-type PKL-PF adhesives can be directly synthesized after the phenolation in the same reactor. Plywood laminated with this adhesive obtains satisfactory strength and low formaldehyde emission. This not only reduces the usage of petroleum-based phenol but also increases the reactivity and applications for hardwood KL.

## 1. Introduction

Lignin, an aromatic polymer with phenylpropane units, accounts for about 30% of the organic carbon in the biosphere [1] and is a promising fossil-fuel alternative for many industrial purposes. It is estimated that the biosphere contains about 3 × 10^11^ tons of lignin, and its annual biosynthetic rate is about 2 × 10^10^ tons [2]. About 70 million tons of lignin are produced annually by the pulp and paper industries, most of which are contributed from pulp mills [3]. Most current industrial applications of technical lignin are lignosulfonate, and the amount is about 1.2 million tons annually [3]. However, about 6–9 million tons of kraft lignin could potentially be extracted from the pulp and paper industries without compromising the energy consumption of the mills if higher-value products can be derived above the fuel value of kraft lignin [4].

Phenol-formaldehyde (PF) resins are among the most important polymeric adhesives used in the wood-based composite panel manufacturing industries to produce products with a high mechanical strength, excellent thermal stability, and great resistance to harsh environments [5]. Phenolic resins are prepared by the reaction of phenol or any substituted phenol with formaldehyde or other aldehydes in acidic conditions to synthesize novolac resins at a molar ratio of F/P < 1 or in alkaline conditions to form resole resins at a molar ratio of F/P > 1. Resoles are often the preferred phenolic resins used in wood adhesives, laminates, fiber-reinforced composites, and many other applications [6]. Currently, phenol is mainly produced from petroleum-based benzene by the cumene hydroperoxide process (the Hock process) [7]. To achieve carbon neutrality, it is logical to use plant-based lignin to substitute petroleum-based phenol because of its phenolic nature and its potential availability in bulk at a low cost compared to phenol. Hence, replacing PF resins with kraft lignin has generated great interest during the past several decades.

A phenol molecule has three reaction sites, two ortho and one para positions, available for the reaction toward formaldehyde during the PF resin formation. However, in lignin, the ortho positions (aromatic C_3_ and C_5_ positions) are often occupied by different numbers of methoxyl groups depending on its composition of monolignol structures. The guaiacyl (G) unit has one methoxyl group occupied in the C_3_ position, whereas the syringyl (S) unit has two methoxyl groups occupied in the C_3_ and C_5_ positions. Hardwood contains both G and S units, whereas softwood contains G units as the majority. The numbers of free ortho positions in lignin vary by the isolation sources and processes. Softwood kraft lignin has typically only 0.3 reactive positions per C_9_ unit [8], and hardwood kraft lignin is expected to have even fewer reactive positions per C_9_ unit due to its lignin structure containing both S and G units. Due to the lower reactivity of kraft lignin toward formaldehyde compared with phenol [9], the replacement ratio is generally limited, and this often results in weaker bonds of wood composite panels when the usage of kraft lignin is increased [7]. The high molecular weight and high polydispersity of kraft lignin also cause viscosity problems in resin formulations [10]. Both result in very limited commercial applications of kraft lignin.

Lignin can be thermally treated with phenol in either acidic or alkaline conditions to increase its reactivity toward formaldehyde, and this process is termed phenolation [11]. The preferred phenolation reaction is under strong acid conditions due to the increased electrophilic aromatic substitution of phenol on the carbocation formed at the C_α_ position of lignin [11], and lignin phenolated under acidic conditions often has higher yields compared to that under alkaline conditions [12]. Phenolation under acidic conditions has proved to be a promising method to increase lignin’s reactivity due to the resulting phenolated lignin having more phenolic hydroxyl groups, and it has been investigated in organosolv lignin [13,14], softwood kraft lignin [15,16], acid-insoluble Klason lignin [17], sodium lignosulphonate [18], biorefinery lignin [19,20,21], etc. However, due to the complex chemical nature of lignin itself, the effective phenolation conditions are different between softwood and hardwood lignin [11]. Under the same phenolation condition, hardwood kraft lignin obtains fewer reactive sites compared to softwood kraft lignin [13]. With the increasing production of hardwood kraft lignin in the world, the successful phenolation conditions of different lignin sources and structures require further optimization for potential applications [22,23].

The objective of this research is to increase the reactivity of hardwood kraft lignin toward resole-type PF adhesive synthesis by phenolation. The phenolation conditions will be optimized to obtain the maximized phenolation yield and suitable lignin fragmentation with minimum acid catalysts and reaction times. The variations in the chemical structures and functional groups were investigated before and after the phenolation. The phenolated hardwood kraft lignin obtained from the optimized process was subsequently used directly without further purification in the resole-type PF adhesive synthesis in a one-pot reaction fashion. Finally, the bonding strengths and properties of the plywood laminated with these adhesives were also evaluated.

## 2. Materials and Methods

### 2.1. Kraft Lignin Preparation

Concentrated mixed-hardwood kraft black liquor, solid content at around 41% (*w*/*w*), was kindly provided by the Chung Hwa Pulp Corporation (Hualien, Taiwan). The mixed hardwood species are mainly *Eucalyptus* spp. and *Acacia* spp. Kraft lignin was precipitated from the concentrated black liquor by adjusting the pH to 9.5 using CO_2(g)_ which is normally readily accessible in pulp mills. The kraft lignin was sequentially washed with 2 N hydrochloric acid and rinsed with deionized water to obtain the crude kraft lignin. The crude kraft lignin was further Soxhlet-extracted with *n*-hexane for 8 h to obtain the extractive-free kraft lignin. This extractive-free kraft lignin (KL) was further vacuum-dried over P_2_O_5(s)_ at room temperature and used for all the analyses and reactions.

### 2.2. Lignin Content and Neutral Sugar Analyses

The lignin content of KL was determined by the Klason lignin method which combines both the acid-soluble and acid-insoluble lignin as the total lignin content [24]. The extinction coefficient used for the calculation of acid-soluble lignin was 110 L g^−1^ cm^−1^ at 205 nm [24]. The residual sugars in KL were determined by the alditol acetate method [25] using the acid-soluble fraction from the lignin content analysis. The residual sugar contents were quantified by gas chromatography with a flame ionization detector (GC-FID, Agilent 7890A, Santa Clara, CA, USA) and a DB-225 GC-column (15 m × 0.25 mm × 0.25 μm).

### 2.3. Uronic Acid, Element, and Ash Content Determination

The uronic acid content in KL was determined by the sulfuric acid-carbazole method [26]. Glucuronolactone (Sigma-Aldrich, St. Louis, MA, USA) was used as the standard, and the optical density was collected at 530 nm. The sulfur, nitrogen, and carbon contents in KL were measured by elemental analysis (Vario EL cube, Elementar Analysensysteme GmbH, Langenselbold, Germany). The ash content of KL was quantified according to the TAPPI standard T211 om-02 (Technical Association of Pulp and Paper Industry, Peachtree Corners, GA, USA) by completely combusting the sample in the crucible at 525 ± 25 °C.

### 2.4. Phenolation of Kraft Lignin

KL was mixed with phenol in the ratio of 3/5 (*w*/*w*), and various amounts of H_2_SO_4_ (1, 5, or 10%, based on the KL weight) were added as the catalyst. The phenol acted as both the reactant and the solvent. Phenolation was carried out at different temperatures (75, 90, 110, or 130 °C) for various numbers of hours (1, 2, or 4 h). To determine the properties of the phenolated kraft lignin (PKL) after the phenolation, PKL was purified from the phenolation solution (PKL/phenol solution) by first being quenched with saturated NaCl_(aq.)_, extracted with ethyl acetate, and precipitated into petroleum ether [27]. The isolated PKL was vacuum-dried over P_2_O_5(s)_ at room temperature and used for the later property analyses. The PKL yield (%) is defined as the percentage of the vacuum-dried weight of PKL over the vacuum-dried weight of KL subjected to phenolation. The optimized phenolation condition was then used for later adhesive synthesis.

### 2.5. Molecular Weight Determination

To evaluate the molecular weight distribution of KL and PKL, the lignin samples were acetylated with acetic anhydride and purified [28]. The molecular weights of the acetylated lignin samples were evaluated by the gel permeation chromatography (Hitachi L-7100, Tokyo, Japan) equipped with a UV–Vis detector (Jasco UV-975, Tokyo, Japan; λ_abs_ = 280 nm) and a PLgel column (3 μm MIXED-E, Agilent, Santa Clara, CA, USA). The solvent was tetrahydrofuran (HPLC grade, Sigma-Aldrich, St. Louis, MA, USA), and various molecular weights of polystyrene (M_w_ = 162 to 10,000 g mol^−1^) were used as the standards to estimate the molecular weights of the acetylated KL and PKL.

### 2.6. UV–Vis and Fourier Transform Infrared Spectrometry

Free phenolic OH, α-carbonyl, and stilbene contents in different lignin samples were estimated by a UV–Vis spectrometer (Jasco V-550, Tokyo, Japan) coupled with the method described in the literature [29]. The α-carbonyl content was estimated from the difference in absorbance at 305 nm between the original and sodium borohydride-reduced lignin solutions. The phenolic OH and stilbene contents of lignin samples were calculated based on the difference in absorbance between the ionized- and the reduced-lignin solutions at 300 and 378 nm. The extinction coefficients were 4100 L mol^−1^ cm^−1^ (300 nm), 9400 L mol^−1^ cm^−1^ (305 nm), and 24,300 L mol^−1^ cm^−1^ (378 nm), respectively [29].

The functional group variations in different lignin samples were analyzed by a Fourier transform infrared spectrometer (FTIR, Bio-Rad FTS-40, USA) coupled with a diffuse reflectance module from 4000 to 400 cm^−1^. The number of scans was 64 with a resolution of 4 cm^−1^.

### 2.7. NMR Analyses of Lignin Samples

Two-dimensional NMR spectra of different lignin samples were recorded on a Bruker AVIII 500 MHz FT-NMR spectrometer (Billerica, MA, USA) at 298 K. The lignin samples were dissolved in DMSO-*d_6_*, and 0.01 M of chromium (III) acetylacetonate was added to the lignin solution as the relaxation reagent [30]. The chemical shifts were calibrated to the DMSO peak (*δ*_C_/*δ*_H_ 39.5/2.5 ppm). The default Bruker “hsqcedetgpsisp2.3” program was used to carry out the heteronuclear single quantum correlation (HSQC) experiment. A total of 16000 scans were collected.

### 2.8. Adhesives Synthesis

The phenol formaldehyde (PF) adhesive was synthesized under the resole-type-resin condition, and the theoretical total solid content in the formulation was 52% (*w*/*w*). All chemicals used were reagent grade. The molar ratio of formaldehyde to phenol (F/P) was 1.9. One molar NaOH_(aq.)_ with a molar ratio of 0.3 to phenol was added slowly into the reaction solution as the catalyst. The solution was stirred at the rate of 200 rpm at 85 °C for 3 h to obtain the PF adhesive.

For the synthesis of kraft lignin phenol formaldehyde (KLPF) adhesive, 3 parts of KL and 5 parts of phenol (*w*/*w*) were used together to replace the total phenol weight in the PF formulation. KL was first dissolved in phenol at 85 °C, and the appropriate amount of formaldehyde with a molar ratio of 1.9 to the phenol used to dissolve KL was added. Subsequently, the NaOH_(aq.)_ (a molar ratio of 0.3 to phenol) was added slowly, and the solution was stirred and reacted at 85 °C for 3 h to obtain the KLPF adhesive.

For the synthesis of phenolated kraft lignin phenol formaldehyde (PKLPF) adhesive, the phenolation and adhesive synthesis were conducted subsequently in the same reactor in a one-pot synthesis fashion. Firstly, KL and phenol (3/5, *w*/*w*) were reacted under the optimized phenolation condition to produce the PKL/phenol solution, and then the acid catalyst (H_2_SO_4_) in the phenolation solution was neutralized by adding the appropriate amount of 1 M NaOH_(aq.)_. The temperature of this neutralized PKL/phenol solution was subsequently adjusted to 85 °C, and the solution was further subjected to adhesive synthesis directly according to the PF formulation and condition. The amount of formaldehyde used in PKLPF adhesive synthesis was solely based on the original amount of phenol used for phenolation with the molar ratio of formaldehyde to phenol being 1.9, and the molar ratio of NaOH to phenol was still kept at 0.3.

At the end of each adhesive synthesis reaction, the reaction solution was cooled down immediately to 25 °C. A viscometer (Model LVT, AMETEK Brookfield, Middleboro, MA, USA) was used to evaluate the viscosities of different adhesives at 25 °C.

### 2.9. Plywood Bonding Strength and Formaldehyde Emission Evaluation

The plywood production was conducted according to the CNS 5808 and CNS 1349 standards [31,32]. Three-layered plywood (3-ply, 45 × 45 cm^2^) with a total thickness of about 7.2 mm was produced using radiata pine veneers. The amount of adhesive in the individual glue line was 170 g m^−2^, and the plywood was hot-pressed at 175 ± 2 °C for 10 min at a pressure of 10 kgf cm^−2^. The plywood was cut into test specimens (80.0 × 25.0 × 7.2 mm^3^) with two 2.5 mm notches equally separated by 25.0 mm on either side of the specimen. Specimens were pre-conditioned at 23 ± 2 °C and 65 ± 5% R.H. (relative humidity) for more than 3 days before the shear strength tests. The specimens were tested in two conditions, normal dry (dry strength) and repetitive boiling (wet strength), according to the CNS 1349 standard [32] using a universal strength test machine (Shimadzu UH-A, Kyoto, Japan) at a loading speed of 1 mm min^−1^. The shear strength of the normal dry test was conducted at 23 ± 2 °C and 65 ± 5% R.H. The specimens for the repetitive boiling test were placed in boiling water for 4 h, followed by drying at 60 °C for 20 h, followed by another 4 h in boiling water, cooled to room temperature by soaking in cold water, and tested for shear strength while the specimens were still wet. At least 24 specimens were tested in either dry or wet strength conditions. According to the CNS 12001 standard [33], the minimum requirements for the dry and wet shear strengths are 1.18 and 0.98 MPa, respectively.

A formaldehyde emission test of the plywood was also conducted according to the CNS 1349 standard [32]. In total, 10 specimens (150.0 × 5.0 × 7.2 mm^3^) without edge-sealing were placed inside a sealed 10 L glass chamber containing a glass dish with 300 mL of de-ionized water in the bottom. After conditioning for 24 h at 20 °C, the concentration of the absorbed formaldehyde in this de-ionized water was quantified by the acetylacetone method. The maximum allowance of the formaldehyde emission for the plywood to be used for indoor application is 0.3 mg L^−1^.

### 2.10. Statistical Analyses

The contents of free phenolic OH, α-carbonyl, and stilbene in different lignin samples were compared all together by multiple means comparisons (Tukey HSD Test, α = 0.05) to test the significant differences among groups. The one-sample *t*-test (α = 0.05) was used to determine whether the shear strengths or the formaldehyde emission of plywood laminated with different adhesives were significantly higher or lower than the required values. All statistical tests were conducted using the SAS software (Ver. 9.3, SAS Institute Inc., Cary, NC, USA).

## 3. Results and Discussion

### 3.1. Basic Properties of the Extractive-Free Kraft Lignin

The yield of this KL was 28.2% (*w*/*w*) based on the solid content of the black liquor. This yield is comparable to the yields (27–35%) from the literature [34,35] in which their lignin samples were precipitated from either kraft or soda-anthraquinone black liquors. The ash, sulfur, nitrogen, and carbon contents of this KL were 3.2, 1.4, 0.1, and 53.9% (*w*/*w*) based on the vacuum-dried KL weight, respectively. Industrial kraft lignin is a product from the kraft pulping process, and it normally contains 2–3% of sulfur varying from 1.5 to 8% [36]. Hence, the sulfur content of our isolated KL is considered low when compared to the usual. The chemical composition of this KL is shown in Table 1. The total lignin and the residual polysaccharide contents of this KL were 87.5% and 6.6% with xylan (3.9%) and glucan (2.0%) being the major residual polysaccharides. Similar total lignin contents were also reported on the kraft lignin from poplar [37] and the alkaline lignin from birch hardwood [20]; hence, this KL was considered good quality and used as the lignin source for the subsequent experiments.

### 3.2. Effects of Phenolation Conditions on the Extractive-Free Kraft Lignin

To seek a way to reduce the molecular weight and to improve the reactivity of the hardwood kraft lignin, this KL was reacted with phenol (KL/phenol = 3/5, *w*/*w*) at various temperatures and times with different percentages of H_2_SO_4_. The favored reaction condition needs to reduce the molecular weight of KL and maximize the phenolation yield with a low reaction temperature and a minimum amount of catalyst to increase the reactivity toward adhesive synthesis. The phenol acts both as the reactant and the solvent to dissolve KL in the reaction so that no extra solvent is needed. The preliminary experiment showed that the KL cannot completely dissolve in phenol if the ratio of KL/phenol is higher than 3/5 (*w*/*w*); hence, the ratio of 3/5 (KL/phenol) was adopted for all experiments.

**Figure 1:**
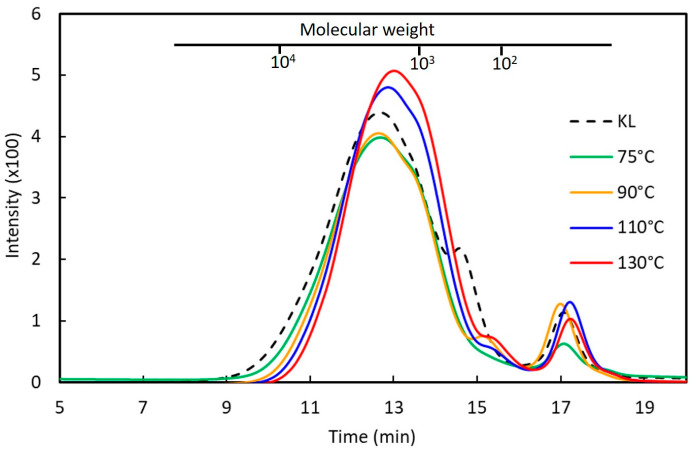
GPC chromatograms of the extractive-free kraft lignin (KL) and the phenolated kraft lignins (PKLs) under different phenolation temperatures. Phenolation condition (KL/phenol = 3/5 (*w*/*w*), 5% H_2_SO_4_, 2 h).

The influence of phenolation temperature on the molecular weight distribution and the yield of the phenolated kraft lignins (PKLs) is shown in Figure 1 and Table 2. The weight-average molecular weight (M_w_) of the original KL is 2181 g mol^−1^ with a polydispersity of 2.60 (Table 2). As the reaction temperature increased from 75 to 130 °C, the M_w_ of PKLs gradually reduced from 1940 g mol^−1^ to 1347 g mol^−1^. The polydispersity of the PKLs also reduced from 2.07 to 1.91, whereas the yields of the PKLs increased from 115 to 126–127%. This indicates that with the increase in the phenolation temperature, the hardwood kraft lignin was fragmented into more uniform and low-molecular-weight PKLs (Figure 1). A similar molecular weight reduction was also reported on the sweetgum biorefinery lignin [15] and pine kraft lignin [16] under a higher phenolation temperature in acidic condition.

**Figure 2:**
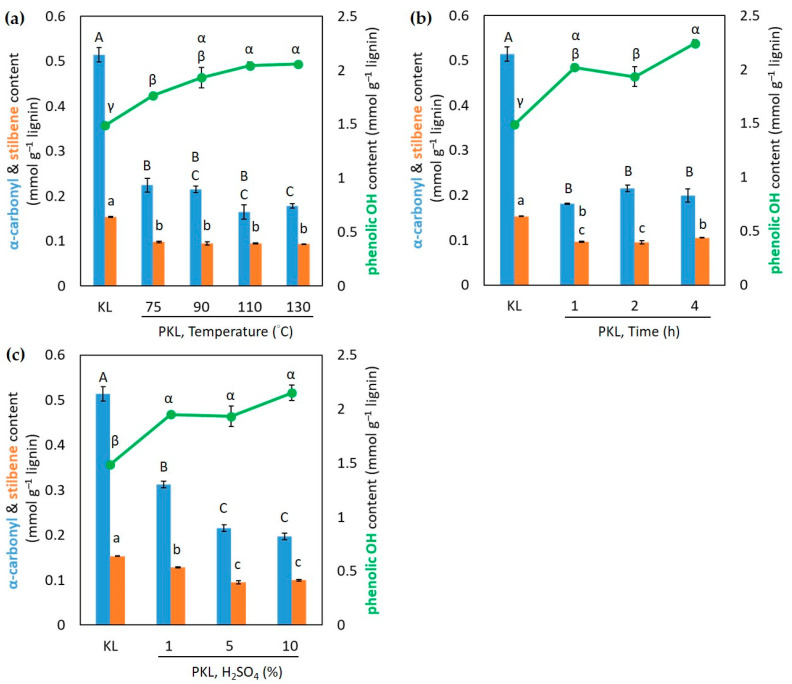
The contents of α-carbonyl, stilbene, and phenolic OH in KL and PKLs under different conditions of phenolation with KL/phenol = 3/5 (*w*/*w*). (**a**) Different temperatures, 5% H_2_SO_4_, 2 h; (**b**) different times of reaction, 5% H_2_SO_4_, 90 °C; (**c**) different amounts of H_2_SO_4_, 90 °C, 2 h. Results are the mean ± SE (n = 3). Different letters inside each graph indicate statistically different under the Tukey HSD test, α = 0.05.

UV spectroscopy is a fast and easily accessible method to quantify the variations in phenolic OH and other functional groups of lignin after phenolation [16]; hence, the contents of α-carbonyl, stilbene, and phenolic OH in KL and PKLs under different conditions of phenolation were estimated by UV spectroscopy. The results indicate that the contents of α-carbonyl and stilbene in PKLs are reduced in any temperature of phenolation compared to that of unreacted KL (Figure 2a), and there is no significant difference between the reaction temperatures. However, the phenolic OH contents gradually increase and there is no significant increase at reaction temperatures higher than 90 °C (Figure 2a). The maximum PKL yield and phenolic OH content were both obtained from the phenolation at 90 °C; hence, this phenolation temperature, 90 °C, was selected as the temperature for the further optimization of other phenolation conditions.

The variations in molecular weight and PKL yield under different reaction times are shown in Table 3. As the reaction time increases from 1 to 4 h, the M_w_ only reduces a little from 1799 to 1703 g mol^−1^. The polydispersity and the maximum PKL yield are still around 2 and 127–128%. As for the changes in the α-carbonyl and stilbene contents in PKLs under different reaction times (Figure 2b), they are all reduced in any given time of reaction compared to that of unreacted KL. The phenolic OH contents of PKLs all increase compared to that of unreacted KL and can be considered to level off after 1 h of phenolation. Prolonging the reaction time to 4 h did not significantly increase the PKL yield; hence, 2 h was selected as the reaction time for the further optimization of different H_2_SO_4_ percentages.

The results from different percentages of the catalyst H_2_SO_4_ (1, 5, and 10%) in phenolation are also listed in Table 3. The M_w_ of PKL is reduced from 1775 g mol^−1^ to 1676 g mol^−1^ with the maximum PKL yield being 127% when using 5% of H_2_SO_4_. The α-carbonyl and stilbene contents are also reduced and level off when the percentage of H_2_SO_4_ is more than 5% (Figure 2c). The phenolic contents in PKLs increase after phenolation, but there are statistically insignificant differences in any tested H_2_SO_4_ percentage (Figure 2c). Considering both the PKL yields and the functional group changes, 5% H_2_SO_4_ is selected for the best condition. Among these tested parameters, including the temperature, reaction time, and percentage of catalyst, the results indicate that the phenolation temperature seems to have a more critical influence on the molecular weight reduction and PKL yield increase compared to the reaction time and H_2_SO_4_ percentage. A similar temperature effect was reported from acidic phenolation on wheat straw lignin from a biorefinery process [21]. However, acid charges were reported to have significant effects on the molecular weight and yield of an acid-phenolated pine kraft lignin [15], and this discrepancy may be due to the different lignin origins.

Considering the results above and the objective of the phenolation, the optimized phenolation condition for this hardwood kraft lignin is heating 3/5 (*w*/*w*) of KL/phenol at 90 °C for 2 h with 5% H_2_SO_4_ as the catalyst. This phenolation temperature (90 °C) is rather low compared to the acidic phenolation condition from the literature which is usually more than 100 °C [15,16,21]. Under this condition, the PKL yield is 127% (*w*/*w*) based on the original KL subjected to the phenolation, and the M_w_ of lignin is reduced from 2181 to 1760 g mol^−1^ with the polydispersity reduced from 2.60 to 2.02 (Table 2). The phenolic OH content of the lignin estimated by UV spectroscopy increased from 1.49 mmol g^−1^ (KL) to 1.93 mmol g^−1^ (PKL) under this reaction condition (Figure 2a), indicating that phenolation creates extra phenolic OH groups in lignin either by adding new phenol groups onto KL or by cleaving KL to create new phenolic OH groups in PKL. Research has suggested that lignin having a reduced and homogenous molecular weight with a high phenolic OH content is essential to produce reproducible and strong thermosetting wood adhesives [9,20,38]. Hence, the PKL produced under this condition can be a good source for the synthesis of the PF adhesive.

### 3.3. Chemical Structure Variation in the Phenolated Kraft Lignin

**Figure 3:**
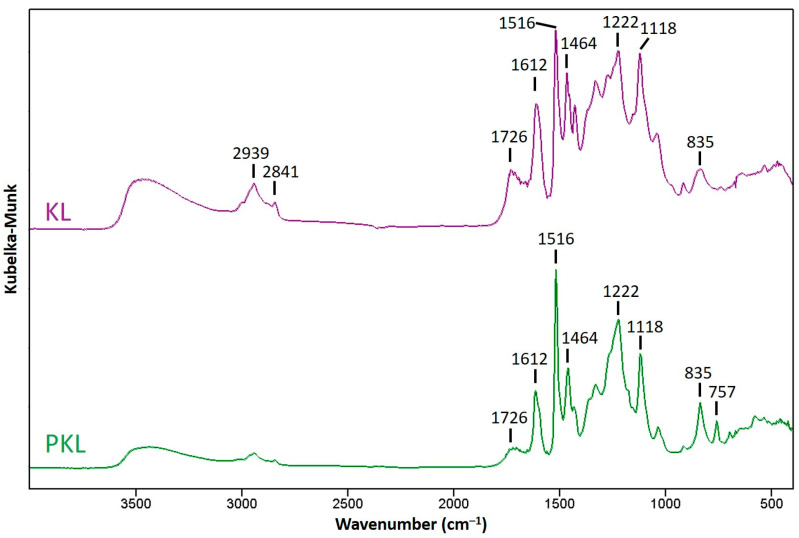
FTIR spectra of KL and PKL under the phenolation condition of KL/phenol = 3/5 (*w*/*w*), 90 °C, 2 h, and 5% H_2_SO_4_.

To further understand the chemical structure changes of lignin after phenolation, the PKLs isolated from the optimized condition were analyzed with FTIR and compared with KL. As indicated in Figure 3, the peak at 1726 cm^−1^ is assigned to C=O stretching [39], and this peak became weaker in PKL, indicating that the number of carbonyl groups in KL was decreased after phenolation. The peaks at 1516 and 1612 cm^−1^ are related to the aromatic skeletal vibration of lignin, and the relative peak ratio of these two peaks in KL represents a typical S/G type hardwood lignin [39]. However, the relative intensity of 1516 cm^−1^ became stronger after phenolation, and this might be attributed to the newly attached phenol in the lignin structure. The peaks at 1464 and 1118 cm^−1^ are relatively stable after phenolation, and they are assigned to the asymmetric C–H deformation of methylene group and aromatic C–H in-plane deformation [9], respectively. The peak at 1222 cm^−1^ increased after phenolation, and the increase in intensity was contributed by the phenolic C–O stretching vibration of newly added phenol groups [9,40]. A similar increase in peak intensity can also be detected at 835 cm^−1^. This peak is assigned to the C–H out-of-plane bending vibration in C_2_ and C_6_ of the S-unit and in all positions of the H-unit [39], and the increased peak intensity of PKL is mainly contributed from the newly attached phenols acting as H-units in PKL. A new peak at 757 cm^−1^ appeared in the FTIR spectrum of PKL, and this peak is from the C–H out-of-plane bending vibration in the substituted phenol caused by the reaction between the ortho and parapositions of phenol and the α-hydroxyl groups of the lignin side chains [41].

**Figure 4:**
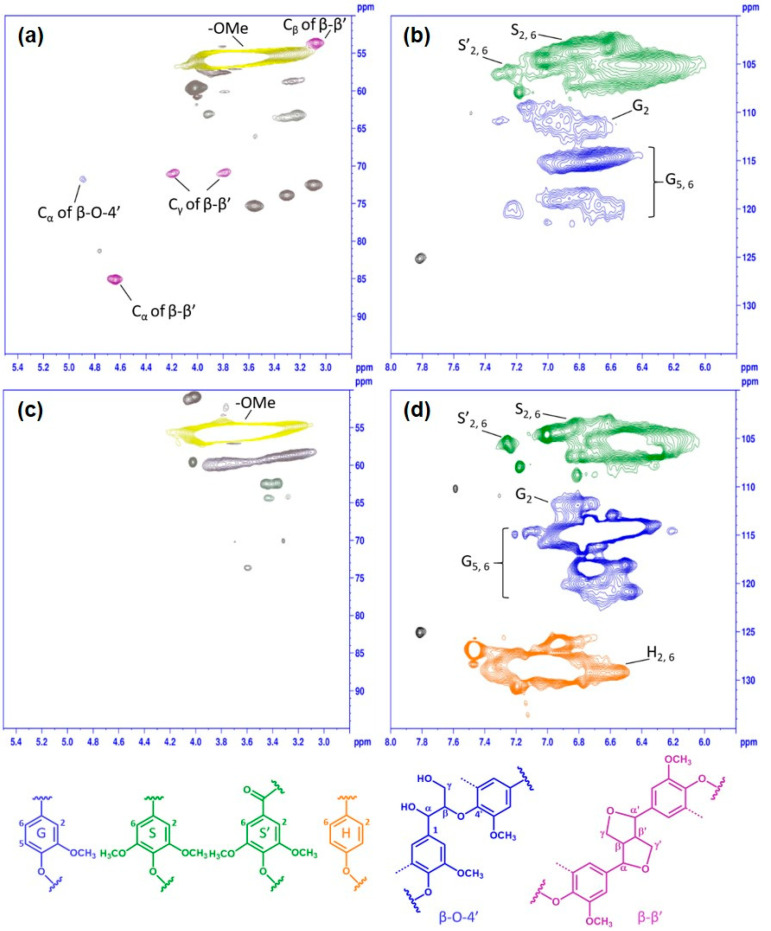
Two-dimensional ^1^H–^13^C HSQC NMR spectra of KL (**a**,**b**) and PKL (**c**,**d**) under the phenolation condition of KL/phenol = 3/5 (*w*/*w*), 90 °C, 2 h, and 5% H_2_SO_4_.

To gain further insight into the chemical structure variations, KL and PKL were subjected to ^1^H–^13^C HSQC NMR analysis (Figure 4). The signals were compared and assigned according to the literature [15,16,21,42,43]. In the side chain region of KL (Figure 4a), the major signals of lignin found were methoxyl groups (–OMe, *δ*_C_/*δ*_H_ 55.7/3.76 ppm), β-aryl ether units (β-O-4’, C_α_–H_α_ at *δ*_C_/*δ*_H_ 71.8/4.89 ppm), and pinoresinol units (β-β’, C_α_–H_α_ at *δ*_C_/*δ*_H_ 85.1/4.64 ppm, C_β_–H_β_ at *δ*_C_/*δ*_H_ 53.5/3.08 ppm, C_γ_–H_γ_ at *δ*_C_/*δ*_H_ 70.8/3.79 and 4.18 ppm). Three cross-peaks of *δ*_C_/*δ*_H_ at 72.5/3.09 ppm (C_2_–H_2_), 73.8/3.30 ppm (C_3_–H_3_), and 75.2/3.55 ppm (C_4_–H_4_) indicating the residual β-D-(1→4) xylan from the lignin–carbohydrate complexes in KL were also detected (Figure 4a). In the aromatic region of KL (Figure 4b), it can be seen that the cross-peaks in the region of *δ*_C_/*δ*_H_ 102–109/6.0–7.4 ppm and *δ*_C_/*δ*_H_ 109–122/6.4–7.4 ppm are due to the S- and G-unit lignin in KL, confirming a typical hardwood lignin.

After the phenolation, the 2D NMR spectrum of PKL showed dramatic changes compared to that of KL. The signals of β-aryl ether units (β-O-4’), pinoresinol units (β–β’) and residual xylan disappeared from the side chain region of PKL after phenolation (Figure 4c), and a new signal at *δ*_C_/*δ*_H_ 50.9/4.02 ppm emerged. An extra strong signal showed up at *δ*_C_/*δ*_H_ 125–133/6.5–7.5 ppm in the aromatic region of PKL (Figure 4d), and this new signal (H_2, 6_) was from the hydroxyphenyl moiety newly attached onto the original KL structures. The signal of the S-unit with α-carbonyl groups (S’_2, 6_) was slightly reduced, indicating the possible reaction of the α-carbonyl groups with phenol. Similar observations were also reported in the acid-catalyzed phenolation process of pine kraft lignin [15,16], bio-refined wheat straw lignin [21] and sugarcane bagasse kraft lignin [44]. In bio-refined wheat straw lignin, phenolation even hydrolyzed more than 50% of the carbohydrates in the lignin sample subjected to phenolation, resulting in a further decrease in aliphatic hydroxyl groups in the lignin sample [21]. Overall, the β-O-4’, β–β’, α-carbonyl structures, and residual carbohydrates of lignin samples were degraded during the phenolation, and the introduced phenol increased the content of hydroxyphenyl unit in the original lignin structures.

### 3.4. Plausible Reactions in Acid-Catalyzed Phenolation on Hardwood Kraft Lignin

**Figure 5:**
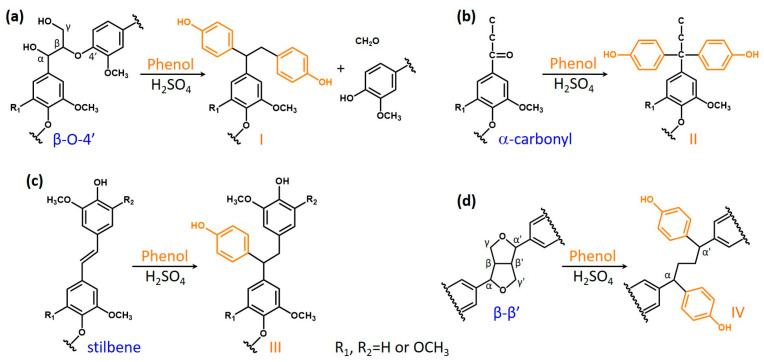
Plausible phenolation mechanism of hardwood kraft lignin. (**a**) β-O-4’ structure, (**b**) α-carbonyl structure, (**c**) stilbene structure, and (**d**) β–β’ structure.

Based on the aforementioned results from the functional group and NMR analyses, and also comparison to the published literature [15,16,45,46,47], the following reactions (Figure 5) might occur in hardwood KL during phenolation. In the β-O-4’ structure (Figure 5a), the C_α_–OH was protonated in acidic conditions, and subsequently converted to a carbocation [45]. The phenol was added to the carbocation on its ortho- or paraposition by electrophilic reaction [46]. Meanwhile, the C_γ_–OH could also be released as a molecule of formaldehyde via β-elimination to form an enol-ether structure under acidic conditions. The subsequent addition of phenol into this enol-ether structure would cleave the β-O-4’ bond of KL and produce the plausible structure I in PKL, and the released formaldehyde could also react with phenol or degraded lignin to form other compounds [15,16]. These would cause the reduction in the β-O-4’ content and molecular weight in PKL (Table 2 and Table 3 and Figure 1 and 4). Similarly, phenol would add to the α-carbonyl and stilbene structures of KL under acidic conditions to form the plausible structures II and III in PKL (Figure 5b and 5c) [27,47], resulting in the reduction in the α-carbonyl and stilbene contents in PKL (Figure 2). As for the β–β’ structure (Figure 5d), the C_γ_– and C_γ’_–OH were eliminated as two formaldehydes to form a conjugated diphenyl-butadiene intermediate at first, and phenols subsequently added to form the plausible structure IV in PKL [15,16], resulting in the reduction in the β–β’ signal in PKL (Figure 4c) and the increase in the hydroxyphenyl moiety in PKL (Figure 4d). The aforementioned emerging signal at *δ*_C_/*δ*_H_ 50.9/4.02 ppm in Figure 4c is actually coming from the C_α_–H_α_/C_α’_–H_α’_ of the plausible structure IV [15,16]. A resinol lignin model compound (β–β’ structure) study further suggested that various naphthalene structures would form under acidic phenolation conditions [48], indicating the great structural complexities of PKL under acidic conditions. In all four types of reactions, phenols were added to the original KL structures, resulting in the increase in phenolic OH contents and yields in PKL. These newly created phenolic OH groups are expected to increase the reactivity of PKL when used in the synthesis of the PF adhesive.

### 3.5. Adhesive and Plywood Property Evaluation

In order to assess the reactivity changes in KL after phenolation, PF, KLPF, and PKLPF adhesives were synthesized accordingly, and three-layered plywood was produced from radiate pine veneers with these three different adhesives. Both dry and wet (repetitive boiling water soaking) bonding tests were conducted to evaluate the bonding strength of these samples. The results indicated that plywood laminated with the PF adhesive obtained 2.07 MPa in dry bonding and 1.46 MPa in wet bonding strengths (Table 4). Both meet the requirements of the regulation: dry strength ≥ 1.18 MPa and wet strength ≥ 0.98 MPa. For plywood laminated with KLPF adhesive (partial phenol replaced with KL without phenolation), the dry bonding strength is 1.12 MPa and the wet bonding strength is 0.75 MPa. Both fail to meet the requirement of regulation, indicating that directly replacing partial phenol with hardwood KL without phenolation for a PF-type adhesive formulation cannot provide the required bonding strength for plywood application. For plywood laminated with the PKLPF-90 adhesive (partial phenol replaced with PKL phenolated at the optimal condition, 90 °C), their dry and wet bonding strengths, 1.71 MPa and 1.10 MPa, both meet the requirements (Table 4). These indicate that phenolation on hardwood KL improved the bonding properties of the PKLPF adhesive required for the plywood application both for dry and repetitive boiling conditions. Another PKLPF-130 adhesive (partial phenol replaced with PKL phenolated at 130 °C) was also tested for the bonding strengths of plywood. This testing group was chosen due to having a comparable phenolic OH content and PKL yield but with a relatively small M_w_ compared to that of the PKL obtained from the optimal phenolation temperature at 90 °C (Table 2 and Figure 2a). Both the dry and wet bonding strengths of the plywood laminated with the PKLPF-130 adhesive meet the requirements for the regulation; however, the viscosity of the PKLPF-130 adhesive (3625 cP, Table 4) was highly viscous and created difficulties in quickly and evenly spreading the adhesives onto the veneer surface. The present industry practice often demands a low-viscosity (< 2000 cP) adhesive formulation [49]; hence, the PKLPF adhesive with a too-small M_w_ in PKL might not be suitable to be used as an adhesive for plywood applications.

Formaldehyde emission has raised public health concerns in recent years due to formaldehyde being known for its carcinogenicity. The formaldehyde emission regulated by CNS 1349 [32] requires the average formaldehyde emission to be no more than 0.3 mg L^−1^ for indoor application. The formaldehyde emission results from all testing groups were all significantly lower than the critical threshold (Table 4). The common hot-press temperatures were in the range of 135–160 °C [14,20,44]. The low formaldehyde emission of all testing groups might be due to the higher hot-press temperature (175 °C) in this study, which promotes the methylene linkages formation of the resin [50]; hence, this reduces the formaldehyde emission of the plywood. The results indicated that plywood laminated with PKLPF adhesives using the current hot-press conditions is suitable even for indoor applications, though plywood laminated with the PF-type adhesive is normally used for outdoor and harsh weather applications. Overall, although the bonding strength of the plywood laminated with the PKLPF-90 adhesive was slightly lower than that of the plywood laminated with the PF adhesive, 37.5% (*w*/*w*) of phenol was replaced with hardwood KL in the PKLPF-90 adhesive compared to the PF adhesive. This partial bio-based phenolic adhesive provides a better bonding strength and lower formaldehyde emission than that of the required regulation. Collectively, phenolation is an effective chemical modification to improve the reactivity of hardwood kraft lignin toward formaldehyde in the synthesis of resole-type PF adhesives.

## 4. Conclusions

The reactivity of hardwood KL toward formaldehyde in the synthesis of resole-type PF adhesives can be increased by phenolation under the suitable condition that is heating three parts of KL with five parts of phenol at 90 °C for 2 h using 5% H_2_SO_4_. This low-temperature phenolation with the catalytic amount of acid can cleave the chemical structures of KL, and phenols are crosslinked onto KL to produce PKL. These one-pot resole-type PF adhesives containing PKL were used for plywood production, and satisfactory plywood bonding strengths and low formaldehyde emissions could be achieved by replacing 37.5% phenol with KL in the original resole-type PF formulation. This not only reduces the usage of petroleum-based phenol but also increases the values and applications of hardwood KL. More in-depth studies on optimizing other parameters such as the reaction sequences, temperatures, catalysts, and the ratios of phenol and formaldehyde of the adhesive formulation are worth investigating to further produce adhesives with diversified properties and performances.

## Figures and Tables

**Table 1:** Chemical composition of the extractive-free kraft lignin (KL).

Composition	Weight Percentage ^2^
Total lignin	87.5 ± 0.3
Acid-soluble	4.9 ± 0.2
Acid-insoluble	82.5 ± 0.2
Residual polysaccharides ^1^	6.6 ± 0.1
Rhamnan	0.4 ± 0.1
Arabinan	0.1 ± 0.1
Xylan	3.9 ± 0.0
Mannan	0.1 ± 0.0
Galactan	0.0 ± 0.1
Glucan	2.0 ± 0.1
Uronic acid	0.6 ± 0.3

^1^ Residual polysaccharide contents are expressed in their polymeric forms. ^2^ Based on the vacuum-dried weight of KL. These values are rounded to the 1st decimal. Results are the mean ± SD (n = 2).

**Table 2:** Molecular weight distributions and yields of the phenolated kraft lignins (PKLs) from different phenolation temperatures.

Sample	Temperature (°C)	M_w_ (g mol^−1^)	Polydispersity (M_w_/M_n_)	PKL Yield ^2^ (%)
KL		2181	2.60	
PKLs ^1^	75	1940	2.07	115
	90	1760	2.02	127
	110	1539	1.94	127
	130	1347	1.91	126

^1^ Phenolation condition (KL/phenol = 3/5 (*w*/*w*), 5% H_2_SO_4_, 2 h). ^2^ Based on KL’s vacuum-dried weight subjected to phenolation.

**Table 3:** Molecular weight distributions and yields of phenolated kraft lignins (PKLs) from phenolation under different times of reaction and amounts of H_2_SO_4_.

Sample	Time (h)	H_2_SO_4_ (%)	M_w_ (g mol^−1^)	Polydispersity (M_w_/M_n_)	PKL Yield ^2^ (%)
KL			2181	2.60	
PKLs ^1^	1	5	1799	2.39	110
	2	5	1760	2.02	127
	4	5	1703	1.95	128
	2	1	1775	2.41	103
	2	10	1676	1.61	121

^1^ Phenolation condition (KL/phenol = 3/5 (*w*/*w*), 90 °C). ^2^ Based on KL’s vacuum-dried weight subjected to phenolation.

**Table 4:** Properties of adhesives and plywood laminated from different adhesives.

Adhesive ^1^	Viscosity (cP)	Dry Strength ^2^ (MPa)	Wet Strength ^2^ (MPa)	Formaldehyde Emission ^2^ (μg L^−1^)
PF	75	2.07 ± 0.04 *	1.46 ± 0.03 *	3.9 ± 0.9 *
KLPF	225	1.12 ± 0.04	0.75 ± 0.02	3.8 ± 0.1 *
PKLPF-90	725	1.71 ± 0.04 *	1.10 ± 0.02 *	1.1 ± 0.3 *
PKLPF-130	3625	1.64 ± 0.06 *	1.06 ± 0.01 *	0.8 ± 0.1 *

^1^ PF, phenol formaldehyde; KLPF, kraft lignin phenol formaldehyde; PKLPF, phenolated kraft lignin phenol formaldehyde. Numbers after PKLPF- indicate phenolation temperature. ^2^ Results are the mean ± SE (n ≥ 24 for dry and wet strength; n = 3 for formaldehyde emission). * Significantly meets the CNS 12001 [33] and CNS1349 [32] regulation (dry strength ≥ 1.18 MPa; wet strength ≥ 0.98 MPa; formaldehyde emission for indoor application ≤ 0.3 mg L^−1^) by one-sample *t*-test (α = 0.05).

## Data Availability

The original contributions presented in the study are included in the article, further inquiries can be directed to the corresponding author/s.
